# Complex Enzyme-Assisted Extraction Releases Antioxidative Phenolic Compositions from Guava Leaves

**DOI:** 10.3390/molecules22101648

**Published:** 2017-09-30

**Authors:** Lu Wang, Yanan Wu, Yan Liu, Zhenqiang Wu

**Affiliations:** School of Biology and Biological Engineering, South China University of Technology, Guangzhou 510006, China; luwang_1987@163.com (L.W.); wuyanan_xixi@163.com (Y.W.); liuyan1994shuimo@163.com (Y.L.)

**Keywords:** guava leaves, enzyme-assisted extraction, phenolics compounds, antioxidant activity, DNA damage protective

## Abstract

Phenolics in food and fruit tree leaves exist in free, soluble-conjugate, and insoluble-bound forms. In this study, in order to enhance the bioavailability of insoluble-bound phenolics from guava leaves (GL), the ability of enzyme-assisted extraction in improving the release of insoluble-bound phenolics was investigated. Compared to untreated GL, single xylanase-assisted extraction did not change the composition and yield of soluble phenolics, whereas single cellulase or *β*-glucosidase-assisted extraction significantly enhanced the soluble phenolics content of PGL. However, complex enzyme-assisted extraction (CEAE) greatly improved the soluble phenolics content, flavonoids content, ABTS, DPPH, and FRAP by 103.2%, 81.6%, 104.4%, 126.5%, and 90.3%, respectively. Interestingly, after CEAE, a major proportion of phenolics existed in the soluble form, and rarely in the insoluble-bound form. Especially, the contents of quercetin and kaempferol with higher bio-activity were enhanced by 3.5- and 2.2-fold, respectively. More importantly, total soluble phenolics extracts of GL following CEAE exhibited the highest antioxidant activity and protective effect against supercoiled DNA damage. This enzyme-assisted extraction technology can be useful for extracting biochemical components from plant matrix, and has good potential for use in the food and pharmaceutical industries.

## 1. Introduction

The fruits of guava (*Psidium guajava* L.) are used as a source of functional beverages due to thier delicious flavor and nutritional value. Besides the fruit, the leaves also have many uses. In Brazil and China, guava leaves are widely applied as a functional herbal tea or a source of beverage and contain various bioactive compounds, including phenolic acids, flavonoids, and polysaccharides [1,2]. Among them, the strong antioxidant activity of polyphenolics may prevent reactive oxygen species (ROS) damage, DNA mutation, diabetes, cardiovascular diseases, and heart disease [3,4]. Moreover, polyphenolics are one of the most abundant bioactive ingredients in GL resources and are considered to be a natural dietary antioxidant due to their potential health benefits and the availability of their raw materials [5].

Many studies have demonstrated that the phenolic compounds of plant matrix usually exist in the soluble free, soluble-conjugated, and insoluble-bound forms [6,7]. Madhujith and Shahidi (2009) confirmed that there are higher amounts of insoluble-bound and soluble-conjugated phenolics than free phenolics in barley [8]. Adom and Liu, (2002) reported that insoluble-bound phenolics made up over 65% of the total phenolics that exist in corn, wheat, rice bran, and tea products [9]. Researches have also confirmed insoluble-bound phenolics are notably difficult to extract due to their interaction with polysaccharides or proteins through O-glycosidic or C-glycosidic bonds in the cell wall. Naczk and Shahidi, (1989) reported that extraction solvents have no significant influence on promoting the release of insoluble-bound phenolics [10]. Many approaches have been used to improve the release of phenolic components from agricultural and sideline products, such as pressurized liquid extraction, supercritical fluid extraction, microwave- or ultrasound-assisted extraction [11,12,13,14], hydrothermal extraction [7], and far-infrared radiation [15]. The advantages of these extraction methods could increase the extraction yield and decrease the extraction time. However, the disadvantages of these methods, including expensive equipment, small scale, and environmental pollution, seriously hindered their application in agricultural byproducts. Consequently, along with the increasing market demand for phenolic extracts, this has prompted the search for new, eco-friendly, and efficient methods of extraction to improve their recovery and bioavailability. Enzyme-assisted extraction has the advantages of large scale, high efficiency, and environmental protection in improving the contents of phenolics from plant matrix. Landbo and Mayer, (2001) confirmed that specific Grindamyl pectinase-assisted extraction have been successfully used to enhance the recovery of polyphenols from blackcurrants [16]. Mathew and Abraham, (2004) also reported that cellulases and feruloyl esterases remarkably promoted the release of phenolic compounds from wheat bran [17]. Zheng et al. (2009) reported cellulases, pectinases, amaylases, hemicellulases, and glucanases can effectively release phenolic compounds from unripe apples [18]. Although the enzyme-assisted approach has largely improved the extraction yield of bioactive compounds of the aforementioned species, there are no reports on the application of this procedure on GL.

The aims of the present work were to: (1) investigate the ability of different enzyme-assisted extraction methods on improving the release of insoluble-bound phenolics from GL; (2) characterize the changes in the compositions and contents of individual phenolics among the free, soluble-conjugate, and insoluble-bound forms from GL after enzyme-assisted extraction; and (3) evaluate the antioxidant activities and protective effect against DNA damage of soluble phenolic extracts from GL after enzyme-assisted extraction.

## 2. Results

### 2.1. Changes of Total Phenolic and Flavonoid Contents with Enzyme-Assisted Extraction

**Figure 1:**
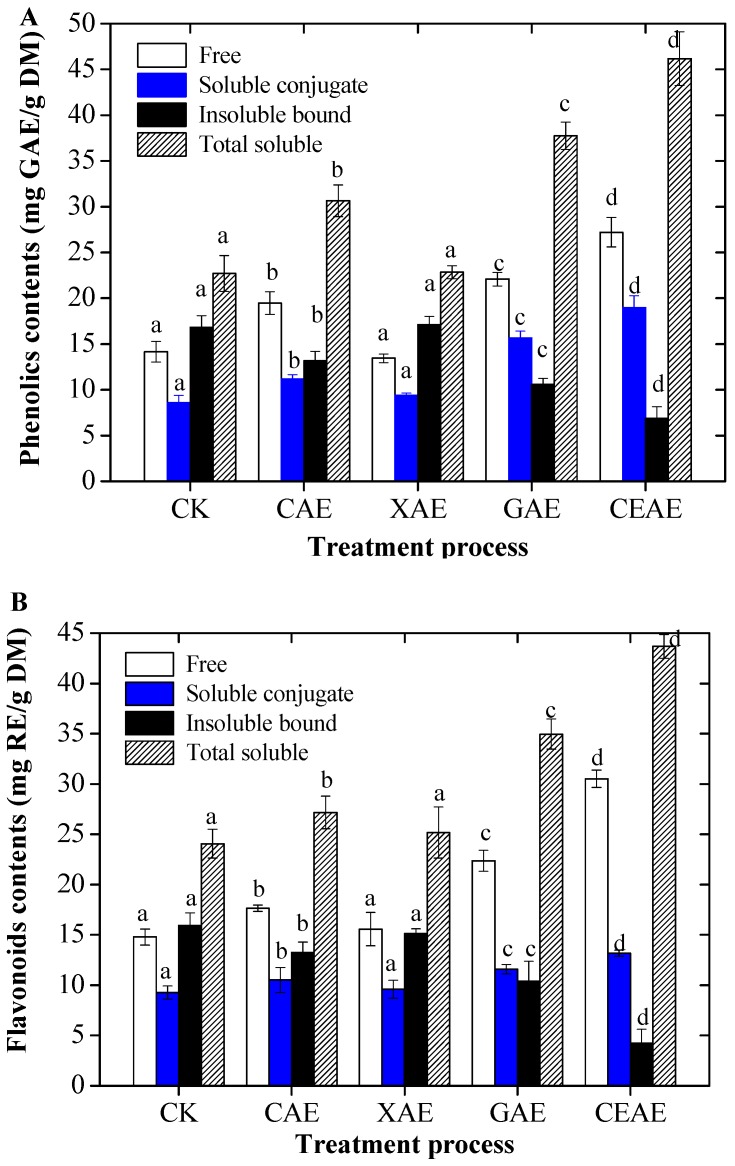
Contents of phenolics (**A**) and flavonoids (**B**), including free, soluble-conjugate, insoluble-bound, and total soluble extracts from guava leaves after different enzyme-assisted extraction. CK, untreated; CAE, cellulase-assisted extraction; XAE, xylanase-assisted extraction; GAE, *β*-glucosidase-assisted extraction; CEAE, complex enzyme-assisted extraction. Values with different letters in each column are significantly different following enzyme-assisted extraction. All experiments were repeated three times and data are expressed as the mean ± standard deviation (SD).

The contents of FP, SCP, IBP, and TSP in GL extracts with different enzyme-assisted extraction methods are shown in Figure 1A. After single cellulase-assisted extraction (CAE), the yields of FP, SCP, IBP, and TSP were 19.5, 13.5, 22.1, and 27.2 mg GAE/g DM, respectively. The FP, SCP, and TSP contents were enhanced by 37.7%, 30.2%, and 34.9% compared to the untreated (CK), respectively. Single xylanase-assisted extraction (XAE) had no significant influence on the phenolic content (*p* = 0.134). Single *β*-glucosidase-assisted extraction (GAE) also significantly increased the contents of FP, SCP, and TSP by 1.6-, 1.8-, and 1.7-fold more than the CK (*p* = 0.000). In addition, complex enzyme-assisted extraction (CEAE) evidently increased the contents of FP, SCP, and TSP by 1.9-, 2.2-, and 2.0-fold compared to the control, and decreased the content of IBP by 59.18%. The ratio of FP to TP was only 35.8% in the CK. However, the ratio increased to 51.4% after CEAE. Importantly, the corresponding IBP decreased from 42.6% in the CK group to 13.0% in the CEAE group.

Figure 1B presents the contents of FF, SCF, IBF, and TSF in the GL extract. After CAE, the amounts of FF, SCF, IBF, and TSF were 17.7, 15.6, 22.4, and 30.5 mg RE/g DM, respectively. The FF, SCF, and TSF were increased by 19.3, 13.4, and 12.9% compared to the CK, respectively. XAE had no significant influence on the TSF content (*p* = 0.211), but GAE significantly increased the contents of FF, SCF, and TSF by 1.5-, 1.3-, and 1.5-fold more than in the untreated group (*p* = 0.001). In addition, CEAE evidently increased the contents of FF, SCF, and TSF by 2.1-, 1.4-, and 1.8-fold more than in the untreated group and decreased the content of IBP by 73.5%. The contribution of FF to the total phenolics increased from 37.0% in the CK group to 63.7% in the CEAE group, while the corresponding IBF decreased from 39.9% in the CK group to 8.8% in the CEAE group.

### 2.2. Changes of Phenolic Compounds with Enzyme-Assisted Extraction

**Figure 2:**
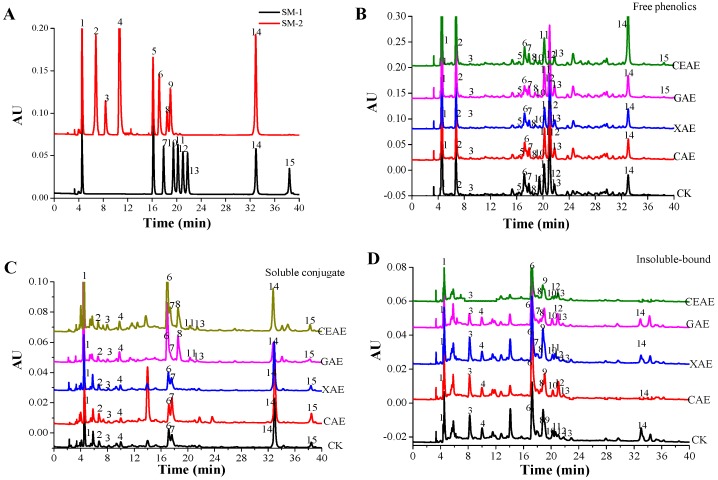
High performance liquid chromatography (HPLC) chromatogram of standard phenolics (**A**); free (**B**); soluble-conjugate (**C**); and insoluble-bound phenolics extracts (**D**) from guava leaves following different enzyme-assisted extraction methods. SM-1, standard mixtures of flavonoids; SM-2, standard mixtures of phenolic acids; CK, untreated; CAE, cellulase-assisted extraction; XAE, xylanase-assisted extraction; GAE, *β*-glucosidase-assisted extraction; CEAE, complex enzyme-assisted extraction. Peaks: 1, Gallic acid, 2, chlorogenic acid, 3, *p*-hydroxybenzoic acid, 4, caffeic acid, 5, rutin, 6, isoquercitrin, 7, *p*-coumaric acid, 8, sinapic acid, 9, ferulic acid, 10, Quercetin-3-*O*-*β*-d-xylopyranoside, 11, Quercetin-3-*O*-α-l-arabinoside, 12, Avicularin, 13, quercitrin, 14, quercetin, 15, kaempferol.

The 15 phenolic compounds including gallic acid, *p*-coumaric acid, chlorogenic acid, *p*-hydroxybenzoic acid, sinapic acid, caffeic acid, ferulic acid, rutin, isoquercitrin, quercetin-3-*O*-*β*-d-xylopyranoside, quercetin-3-*O*-*α*-l-arabinoside, avicularin, quercitrin, quercetin, and kaempferol in free, soluble-conjugate and insoluble-bound fractions from GL after enzyme-assisted extraction were analyzed (Figure 2A–D). Based on the HPLC analysis, the compositions of three form phenolic fractions were similar after enzyme-assisted extraction, while their contents were evidently different (*p* < 0.05). From Figure 2B,C and Table 1, it can be found that quercetin-3-*O*-*β*-d-xylopyranoside and kaempferol were present only in the soluble free form, while chlorogenic acid, rutin, sinapic acid, avicularin, and quercitrin rarely exist as the soluble-conjugate form. Most of the determined phenolics were present in free, soluble-conjugate, and insoluble-bound forms (Figure 2B–D). After CAE, individual phenolics contents from GL extracts were slightly increased with the exception of syringic acid and ferric acid, which did not change significantly (*p* = 0.39). Individual phenolics contents from the GL extracts following XAE did not evidently change (*p* = 0.43). Treatment with *β*-glucosidase can also distinctly increase the total soluble contents of individual phenolics, especially for gallic acid and quercetin, which were enhanced by 36.4% and 156.1%, respectively, while exceptions included rutin, avicularin, and quercetin-3-*O*-*α*-l-arabinoside, which decreased by 72.2%, 72.4%, and 21.8%, respectively. The increases in the total amount of each phenolic compound from GL extracts after CEAE were as follows: gallic acid, 57.0%; chlorogenic acid, 37.7%; *p-*hydroxybenzoic acid, 87.2%; caffeic acid, 86.7%; *p*-coumaric acid, 74.4%; quercetin-3-*O*-*β*-d-xylopyranoside, 35.9%; quercitrin, 25.4%; quercetin, 250.8%; and kaempferol, 119.0%, while the four phenolics that showed declines in total contents were syringic acid, 67.5%, rutin, 90.0%, quercetin-3-*O*-*α*-l-arabinoside, 82.4%, and avicularin, 92.3%.

### 2.3. Changes of Bioactivity with Enzyme-Assisted Extraction

#### 2.3.1. Antioxidant Activity

The antioxidant capacity of FP, SCP, IBP, and TSP extracts from GL following enzyme-assisted extraction were evaluated by three antioxidant modes, including scavenging activity of ABTS^+^, DPPH radical, and FRAP assays. The results are shown in Table 2.

After CAE, the ABTS values of FP, SCP, IBP, and TSP were 23.5, 9.5, 16.8, and 33.0 mmol TE/g DM, respectively. CAE increased the FP, SCP, and TSP ABTS values, by 13.9%, 46.3%, and 21.7%, respectively (*p* < 0.05). However, XAE did not significantly increase the soluble phenolics (*p* = 0.45). In contrast, there is a slight decline in the soluble phenolic ABTS value which may be because several phenolics have been hydrolyzed into other chemicals due to high temperature treatment, such as with syringic acid [19]. After treatment with *β*-glucosidase, the ABTS values of FP, SCP, and TSP were fortified by 40.8%, 66.3%, and 46.9%, respectively. In addition, CEAE evidently increased the ABTS values of FP, SCP, and TSP by 1.8-, 2.7-, and 2.0-fold more than with the CK.

After CAE, the DPPH values of FP, SCP, IBP, and TSP were 17.6, 11.5, 18.2, and 29.1 mmol TE/g DM, respectively. FP, SCP, and TSP DPPH values were significantly increased by 14.6%, 43.3%, and 24.4%, respectively (*p* < 0.05). However, XAE showed no significant change in the soluble phenolics (*p* > 0.05). GAE fortified the DPPH values of FP, SCP, and TSP by 70.2%, 69.4%, and 69.9%, respectively. Interestingly, CEAE evidently enhanced the DPPH values of FP, SCP, and TSP by 2.3-, 2.2-, and 2.3-fold more than with the CK.

Antioxidant activity of GL after enzyme-assisted extraction was also evaluated by FRAP assays. After CAE, the FRAP values of FP, SCP, IBP, and TSP were 94.8, 49.7, 50.5, and 144.4 μmol Fe(II)SE/g DM, respectively. CAE significantly promoted the FP, SCP, and TSP FRAP values by 13.2%, 14.0%, and 13.5%, respectively (*p* < 0.05). However, XAE did not significantly increase the soluble phenolics (*p* = 0.61). Through treatment with *β*-glucosidase, the FRAP values of FP, SCP, and TSP were enhanced by 33.6%, 37.7%, and 35.0%, respectively. Importantly, CEAE evidently increased the FRAP values of FP, SCP, and TSP by 1.9-, 1.8-, and 1.9-fold more than with the CK. The FRAP assays were consistent with the above tested results of ABTS and DPPH.

#### 2.3.2. Protection Effect against DNA Damage

**Figure 3:**
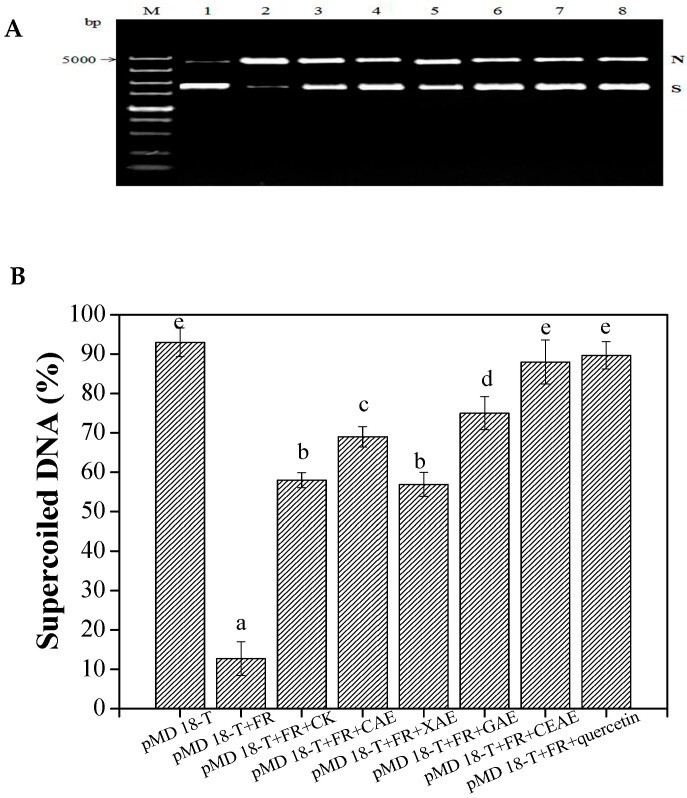
Protective effects (**A**,**B**) of soluble phenolic extracts following different enzyme-assisted extraction methods against DNA oxidation inducted by Fenton’s reagent (FR). The gel was visualized under UV-transilluminator using the Gel Doc XR system (Bio-Rad, Hercules, CA, USA). M: 5000 bp DNA marker; Lane 1: pMD 18-T+ PBS solution; lane 2: pMD 18-T+FR; lane 3: pMD 18-T+FR + CK; lane 4: pMD 18-T + FR + CAE; lane 5: pMD 18-T+ FR + XAE; lane 6: pMD 18-T+ FR + GAE; lane 7: pMD 18-T+ FR + CEAE; lane 8: pMD 18-T+ FR + quercetin. Values are mean ± standard deviation (*n* = 3). N, Nicked DNA; S, supercoiled DNA; FR, Fenton’s reagent; CK, untreated; CAE, cellulase-assisted extraction; XAE, xylanase-assisted extraction; GAE, *β*-glucosidase-assisted extraction; CEAE, complex enzyme-assisted extraction. Different letters (a–e) mean statistically significant differences at *p* < 0.05.

From Figure 3A, the soluble phenolic extracts from GL following different enzyme-assisted extraction methods had significant preventative effects on supercoiled DNA strand scission (*p* < 0.05). In particular, the supercoiled DNA had been almost completely split into the nicked DNA form when mixed with only the plasmids pMD-18T and Fenton’s reagent. The positive control, quercetin, showed a good protective effect against hydroxyl radical-induced DNA supercoiled strain breakage. From Figure 3B, the SP extracts of GL following CAE showed 59.0 ± 0.3% supercoiled DNA, while treatment with *β*-glucosidase exhibited a greater protection of supercoiled DNA (75.7 ± 1.4%) than in the phenolics group without any additions (*p <* 0.01). Treatment with xylanase exhibited no significant improvement on the protection of supercoiled DNA compared to the phenolics group without additions (*p* = 0.27). However, CEAE resulted in the supercoiled DNA of 89.0 ± 2.2%, which displayed no evident differences in comparison with the blank (only plasmid pMD-18T with PBS buffer) and quercetin treatment groups (the positive control). Moreover, SP extracts of GL treated with the complex enzymatic extraction were obviously stronger than those of GL treated with a single enzymatic extraction for protective effects against DNA damage (*p <* 0.05).

## 3. Discussion

### 3.1. Enzyme-Assisted Extraction Action on Total Soluble Phenolics and Soluble Flavonoids Contents

Soluble phenolics can easily be released from the plant matrix, while the extraction of insoluble-bound phenolic compounds is difficult. Phenolic compounds in the insoluble-bound form are covalently bound to plant cell wall structural elements such as cellulose, hemicellulose, and structural protein or polysaccharides [20]. The present study confirmed that xylanase-assisted extraction has no significant effect on improving the yields of the soluble phenolics and flavonoids (free and conjugate form). However, cellulase- or *β*-glucosidase-assisted extraction increases the release of insoluble-bound phenolics and flavonoids. In addition, complex enzyme-assisted extraction is a higher-efficiency method to improve the release of the insoluble-bound phenolics and obtain the highest content of quercetin and kaempferol than a single enzyme-assisted extraction. As we all know, the absorption efficiency in human intestines and the bioactivities of quercetin were much higher than with its glycoside derivatives [21,22,23]. Treatment with *β*-glucosidase, cellulase, and complex enzymes caused the disruption of the bonds between the phenolics and the cell wall components of the GL matrix; thus, the insoluble-bound phenolics were released into soluble forms (Table 2). The results were consistent with the report of Liu et al. (2017) [19]. Similarly, the enzymatic release of phenolic compounds from currant pomace using commercial protease and pectinase yielded higher contents of phenolics. In our study, complex enzyme-assisted extraction is more effective than use of a single enzyme-assisted extraction, which is consistent with previous studies [19,24]. This may be observed because GL is comprised of many flavonoids in the glycosidic bond or OH ground form attached to the cell wall, polysaccharides, or proteins. Many reports have confirmed that *β*-glucosidase and cellulase can more efficiently disrupt the bonds (glycosidic bonds or –OH) [18,19]. However, xylanase may play a few roles in the rupture of the bonds between the phenolics and the cell walls of the plant matrix. Wang et al. (2016) have confirmed that the phenolic contents of GL were enhanced by solid-state co-fermentation with *Monascus anka* and *Saccharomyces cerevisiae*, but the relationship was not clear between the change of free, conjugated, and insoluble-bound phenolics and the release of insoluble-bound phenolics and the enzymatic action during fermentation [5]. Therefore, it is important to perform a comprehensive evaluation of the phenolic profile in GL, including the change of the free, conjugated, and insoluble-bound phenolics under specific enzyme-assisted extraction.

### 3.2. Enzyme-Assisted Extraction Action on the Phenolic Compositions

Generally, CAE increased the soluble contents of individual phenolics compounds from GL, with the exception of syringic acid, which significantly declined, possibly due to degradation upon exposure to high temperatures [19]. GAE may not only promote the release of phenolic compounds, but it can also convert flavonoid glycosides into aglycones [5,25]. GAE released 199.1 mg/100 g DM of quercetin from GL, while CEAE released 258.9 mg/100 g DM, which was 3.5 times that of the control. The great enhancement of quercetin contents was due to the interaction between the enzymatic effects from plant cell wall damage and the biotransformation of quercetin-3-*O*-*α*-l-arabinoside and avicularin. Interestingly, CEAE brought about a larger release of individual phenolics than treatment with a single enzyme, and the soluble free phenolics were greater than the soluble-conjugate and insoluble-bound forms in GL extract, which differs from the reports of Liu et al. (2017) [19]. Based on the result from Table 2, it can be concluded that the increases in the phenolic compounds are mainly due to the release of the insoluble-bound phenolics, such as gallic acid, *p*-hydroxybenzoic acid, caffeic acid, rutin, *p*-coumaric acid, isoquercitrin, quercetin-3-*O*-*β*-d-xylopyranoside, quercitrin, and quercetin. Alrahmany et al. (2013) found that both cellulase and *α*-amylase treatment released more soluble-conjugate than free phenolic acids from oat bran, including ferulic, coumaric, caffeic and vanillic acids, and the amount released significantly differed between the tested enzymes [26]. To summarize, the manner in which some individual phenolics were released differed, as they were differently subjected to the attachments of cell wall components, as well as the specificity of the enzymes.

### 3.3. Enzyme-Assisted Extraction Action on Bioactivity

The potential health benefits of phenolic compounds from tea products or natural cereals are mainly attributed to their strong antioxidant activity. Due to the limitations of a single antioxidant property test to reflect the antioxidant capacity of the samples, it was necessary to implement multiple antioxidant activity assays [27]. The antioxidant activities of different forms of phenolics in the GL extracts were measured. These confirmed that single enzymatic treatment with cellulase or *β*-glucosidase significantly increased the scavenging activity of ABTS^+^, DPPH radical, and FRAP activity. Moreover, the three methods of measuring antioxidant activity gave similar results and were consistent with the change in phenolic contents of GL after enzyme-assisted extraction. Several researchers have reported that there is a positive correlation between antioxidant activity and the contents of phenolics and flavonoids [19,28]. Alrahmany and Tsopmo, (2012) found that the free ORAC activity of oat bran following three different enzymatic treatments can be increased, due to the promoted release of the phenolic contents [29]. Liu et al. (2017) also confirmed that the FRAP and ORAC activities of rice bran were enhanced by complex enzymatic treatment [19]. However, little information has been available on the ability of enzyme-assisted extraction methods to improve the antioxidant activity of soluble phenolics from GL. The present work has confirmed CEAE can significantly enhance the antioxidant activity of GL extracts.

Free radicals are known for damaging DNA strands, which might ultimately lead to cytotoxicity, mutagenesis, and carcinogenesis [30,31]. Hydroxyl radicals generated by the Fenton’s reagent reaction can cause breakage of supercoiled DNA strands and an increase in the formation of the nicked DNA [32]. In the present work, due to the increase of soluble phenolic/flavonoids compounds from GL following cellulase or *β*-glucosidase processing, the SP extracts showed higher protective effects for supercoiled DNA than the phenolics group without additions. However, the SP extracts of GL following complex enzymatic treatment showed the highest inhibition of peroxyl and hydroxyl radical-induced DNA supercoiled strand breakage. According to the HPLC analysis, the contents of quercetin and TSP of GL extracts following complex enzymatic processing were quite evidently enhanced (*p* < 0.01). Chandrasekara and Shahidi, (2011) have confirmed phenolics can significantly prevent supercoiled DNA strand scission [33]. Kim et al. (2010) have reported that flavonoid aglycones possessed an evidently higher protective capacity for supercoiled DNA than flavonoid glycosides [34]. Singh et al. (2010) also verified that fermented legume extracts showed higher DNA damage protection than unfermented legume extracts due to the higher contents of phenolics and flavonoids. Thus, the higher accumulation of soluble phenolics and quercetin were conducive in enhancing the protection of supercoiled DNA [35].

## 4. Materials and Methods

### 4.1. Chemicals and Reagents

All of phenolics standards, Folin–Ciocalteu’s phenol reagent, potassium persulfate, FeCl_3_, Trolox, ABTS, 2,4,6-tripyridyl-Striazine (TPTZ), and DPPH were all purchased from Sigma-Aldrich (St. Louis, MO, USA, *>*99.5%). Formic acid and acetonitrile solvents were purchased from Fisher Scientific (HPLC grade, 99.9%, Waltham, MA, USA). The Takara MiniBEST Plasmid Purification Kit and the pMD-18T plasmid DNA were also purchased from Takara Biotechnology Co., Ltd. (Dalian, China). Cellulase (E.C. 3.2.1.4, 8000 U/g) and xylanase (E.C. 3.2.1.8, 8000 U/g) both from *Aspergillus niger*, *β*-glucosidase (E.C. 3.2.1.21, 8000 U/g) from *Trichoderma reesei*, and complex enzymes (including cellulase, xylanase, and *β-*glucosidase) were purchased from Youtell Biochemical Co., Ltd. (Shanghai, China).

### 4.2. Enzyme Pre-Treatment

The enzymatic reaction system including 5 g of dried and ground GL substrate and 20 mL H_2_O (adjusted to pH = 5.0 using 0.02 M citric acid), was incubated for 12 h at 50 °C with 0.5 g of single enzyme (cellulase, xylanase, or *β*-glucosidase) or 1.5 g of complex enzymes mixture (cellulase:xylanase:*β*-glucosidase; 1:1:1), respectively. Enzymatic treatment was performed in triplicate. After completion of the reaction, all samples were maintained at oven for 20 min at 80 °C to inactivate these enzymes. GL samples treated with different enzymes were collected and dried for 15 h at 60 °C to remove the water.

### 4.3. Extraction of Free Phenolic Fractions

The free phenolic fraction was extracted according to the method with slight modifications [36]. In brief, 1 g of the above treated GL powder was extracted three times with 70% methanol at a ratio of 1:10 (*w*/*v*). For each extraction, the mixture was kept in a 40 °C water bath for 1 h. The filtrate was extracted three times with 70 mL of ethyl acetate via liquid-liquid stratification [19]. After removal of the ethyl acetate, the extract was redissolved in 5 mL of 50% methanol (*v*/*v*).

### 4.4. Extraction of Soluble-Conjugate Phenolics

The soluble-conjugated phenolic fraction was extracted from all GL samples according to the methods described by Bei et al. (2017) with modifications [36]. The conjugated phenolic fraction was extracted from the water phase after the ethyl acetate extraction of the free phenolic fraction. The water phase was hydrolyzed with 40 mL of 2 M NaOH for 4 h and acidified with 12 M HCl to pH 2.0. The hydrolysate was extracted three times with 70 mL of ethyl acetate via liquid-liquid stratification [5,37]. The ethyl acetate fractions were evaporated under vacuum at 45 °C until dry, the extract was redissolved in 5 mL of 50% methanol (*v*/*v*).

### 4.5. Extraction of Insoluble-Bound Phenolics

Insoluble-bound phenolic fraction was extracted according to the methods described by Wang et al. (2017) [38]. The dried leaf residue (0.5 g) from the above free phenolics extraction was hydrolyzed directly with 50 mL of 2 M NaOH at room temperature for 4 h. The mixture was then acidified with 12 N·HCl to pH 2.0. The remaining mixture was then extracted three times with 70 mL of ethyl acetate [19,20]. The ethyl acetate fractions were evaporated under vacuum at 45 °C until dry. The insoluble-bound phenolics were dissolved by adding 5 mL of 50% methanol (*v*/*v*). All phenolics extracts were stored at −20 °C before analysis.

### 4.6. Determination of Phenolic Content

The free, soluble-conjugated, and insoluble-bound phenolics from GL extracts were determined according to the reported method with a minor modification [38]. Briefly, 100 μL of phenolics extracts were mixed with 30 μL of Folin–Ciocalteu reagent and 150 μL of 20% Na_2_CO_3_ solution. After incubation for 30 min at 30 °C in the dark, the absorbance was measured using a SpectraMax Gemini microtiter plate reader (Molecular Devices, Sunnyvale, CA, USA) at 760 nm. Gallic acid (10–100 μg/mL) was used as the reference standard (R^2^ = 0.9995). Phenolics contents were expressed as mg gallic acid equivalents (GAE)/g sample in dry mass (DM) (mg GAE/g DM). Samples were determined in triplicate.

### 4.7. Determination of Flavonoids Content

The flavonoid content was determined using the by the AlCl_3_ colorimetric method [39]. Briefly, 0.1 mL of the above extract was placed in a 2-mL Eppendorf tube. A 70% methanol solution was added to make a 0.5 mL solution, and then, 30 μL of 5% NaNO_2_ solution (*w*/*v*, Tianjin, China) was added. After being kept at room temperature for 5 min, 30 μL of 10% AlCl_3_ solution was added, and the mixture was allowed to stand for another 6 min. Next, a total of 0.2 mL of 1 M NaOH was added, and the total volume was increased up to 1 mL using 70% methanol solution. The solution was thoroughly mixed again and allowed to stand for 30 min at 35 °C. The absorbance was read at 510 nm. Rutin (10–100 μg/mL) was used as a reference standard (R^2^ = 0.9995). The flavonoid content was expressed as mg rutin equivalents (RE)/g sample in dry mass (DM) (mg RE/g DM). All of samples were measured in triplicate. 

### 4.8. HPLC Analysis

The HPLC system was comprised of a Waters e2695 HPLC system equipped with a Waters SunFire^TM^ C_18_ column (250 mm × 4.6 mm, 5 μm, Waters, Milford, MA, USA) as well as a Diode Array Detector (DAD, Waters 2998, Milford, MA, USA). The mobile phase consisted of 0.1% formic acid aqueous solution (*v*/*v*, solution A) and acetonitrile solution (solution B) in the gradient elution at 0.8 mL/min with time-course increasing of solution B to 15% B for 0–5 min, 15–20% B for 5–10 min, 20–25% B for 10–20 min, 25–35% B for 20–30 min, 35–50% B for 30–40 min, 80% B for 40–50 min, and 15% B for 50–55 min. The column temperature was set at 30 °C. The detected wavelength was set at 280 nm. Before HPLC analysis, the samples were filtered through a 0.25-μm membrane filter (Millipore, Billerica, MA, USA). Accurate amounts of standard phenolics were added to GL extracts after enzymatic hydrolysis, and they were extracted as described in Section 4.3, Section 4.4 and Section 4.5. As calculated according to the amount found and amount added, the recovery rate of these phenolics ranged from 95.31% to 101.07% (Table 3). The contents of individual phenolics were expressed as milligram per 100 g DM of GL samples. 

### 4.9. Evaluation of Antioxidant Activity

#### 4.9.1. ABTS Radical Cation (ABTS^+^) Scavenging Activity

The ABTS assay was performed according to the procedure described by Sasipriya & Siddhuraju, (2012) [40]. ABTS^+^ was formed by oxidation of ABTS (7 mM) with K_2_S_2_O_8_ (2.45 mM) after incubation in the dark at room temperature for 16 h. Then, it was diluted with ethanol to obtain an absorbance of 0.70 at 734 nm before use. 100 μL dilution of the above sample extracts were added to 400 μL of the diluted ABTS^+^ solution and mixed thoroughly. The reactive mixture was kept in the dark at room temperature for 30 min, and the absorbance was subsequently recorded at 734 nm. The ABTS^+^ scavenging activity values were expressed in mmol Trolox equivalents (TE)/g sample in DM and were derived from a standard curve.

#### 4.9.2. DPPH Radical Scavenging Activity

The DPPH assay was conducted based on the method described by Hammi, Jdey, Abdelly, Majdoub, & Ksouri, (2015), with slight modifications [41]. Briefly, 50 μL of dilution of the above extracts was mixed with 400 μL of a DPPH-methanol solution (100 μM). The reaction was conducted at 25 °C for 30 min in the dark. The absorbance was measured at 517 nm (A_517_). The DPPH radical scavenging activity values, expressed in mmol Trolox equivalents (TE)/g sample in DM (mmol TE/g DM), were derived from a standard curve.

#### 4.9.3. Ferric Reducing Antioxidant Power (FRAP) Assay

The FRAP assay was measured according to the method reported by Liu et al. (2017) [19]. The fresh FRAP reagent was prepared using 50 mL of 0.3 M acetate buffer (5.1 g sodium acetate in 20 mL CH_3_COOH, pH 3.6), 5 mL of 20 mM FeCl_3_ solution and 5 mL of TPTZ solution (10 mM TPTZ in 40 mM HCl), which was warmed to 35 °C before use. The above extract was diluted with distilled water, and then 30 μL of diluted extracts were mixed with 900 μL of FRAP reagent (freshly prepared). After incubation for 30 min in the dark at room temperature, the absorbance of the reaction mixture was read at 593 nm. The FRAP values, expressed in μmol ferrous sulfate equivalents Fe(II)SE/g sample in DM (μmol Fe(II)SE/g DM), were derived from a standard curve.

### 4.10. Inhibition of Supercoiled DNA Strand Breakage

The inhibitory activity of soluble phenolic extracts from GL following different enzymatic hydrolysis against supercoiled form DNA scission induced by Fenton’s reagent was evaluated based on the method of Liyana-Pathirana and Shahidi, (2006), with slight modifications [42]. Briefly, 10 μL of soluble phenolics extracts (2 mg/mL) was mixed with 5 μL of pMD 18-T plasmid DNA (200 ng/μL) and 10 μL of Fenton’s reagent (50 mM ascorbic acid, 80 mM FeCl_3_, and 30 mM H_2_O_2_). The mixture was later incubated in the dark for 30 min at 37 °C. Quercetin was used as a positive control, and phosphate buffer saline (PBS) instead of the sample as the blank control. The DNA samples were electrophoresed using 0.8% agarose gel with 10% (*v*/*v*, μL/mL) GoldView stain (Solaibio Science and Technology, Co., Ltd., Beijing, China), and the DNA bands were visualized under transillumination of UV light using the Gel Doc XR system (Bio-Rad, Hercules, CA, USA). The intensity of the DNA bands was analyzed using Quantity One software (Version 4.6.2, Bio-Rad, Hercules, CA, USA). The relative percentage of the supercoiled DNA was used to evaluate the DNA protective effect of soluble phenolics extracts of GL on the basis of the following Equation (1) [33]:

(1) SupercoiledDNA(%)=ISIS+IN×100

where *I_s_* is the intensity of the supercoiled DNA, and *I_N_* is the intensity of the nicked DNA.

### 4.11. Statistical Analysis

All experiments were repeated three times, and data are expressed as the mean ± standard deviation (SD). Data were analyzed by the SPSS statistics 13.0 software (StatSoft, Tulsa, OK, USA). A one-way analyses of variance (ANOVA) method was applied to evaluate the significance of the differences among the mean values of the test levels. Differences with *p* < 0.05 and <0.01 were considered to be significant and highly significant, respectively. 

## 5. Conclusions

Overall, single xylanase-assisted extraction did not change the composition and yield of soluble phenolics from GL. Single cellulase or *β*-glucosidase-assisted extraction significantly increased the soluble phenolics content of GL. Meanwhile, not only did complex enzyme-assisted extraction enhance the yield of soluble phenolics from GL, but also it can convert the flavonoid glycosides into flavonoid aglycones (quercetin and kaempferol) with stronger antioxidant activity. Additionally, soluble phenolic extracts of GL after complex enzyme-assisted extraction exhibited the highest antioxidant activity and protective effect against oxidative damage of DNA induced by Fenton’s reagent. The results of this study provide useful information for processing GL into polyphenol-based food sources or tea products with increased health benefits.

## Figures and Tables

**Table 1:** Changes in individual phenolics in GL extract following enzymatic treatment of the free, soluble-conjugate, insoluble bound, and total soluble phenolics fractions.

Analytes	Stage	Free (mg/100 g DM)	Soluble-Conjugate (mg/100 g DM)	Insoluble-Bound (mg/100 g DM)	Total Soluble (mg/100 g DM)
Gallic acid	CK	161.1 ± 1.11a	118.3 ± 1.32a	351.5 ± 2.63a	279.5 ± 2.33a
	CAE	173.0 ± 1.31b	131.4 ± 1.81b	259.6 ± 1.53b	304.4 ± 3.12b
	XAE	163.4 ± 2.21a	117.5 ± 2.12a	348.8 ± 2.01a	280.9 ± 4.33a
	GAE	235.3 ± 1.90c	145.7 ± 1.31c	245.7 ± 1.41c	381.1 ± 3.21c
	CEAE	276.6 ± 2.62d	162.2 ± 1.71d	71.36 ± 1.45d	438.8 ± 4.33d
Chlorogenic acid	CK	25.8 ± 0.31a	26.3 ± 0.40a	N.D.	52.1 ± 0.71a
	CAE	31.1 ± 0.25b	29.4 ± 0.31b	N.D.	60.5 ± 0.56b
	XAE	23.7 ± 0.61b	27.4 ± 1.22b	N.D.	51.8 ± 1.83a
	GAE	36.5 ± 0.37b	30.7 ± 0.42b	N.D.	67.2 ± 0.73b
	CEAE	41.4 ± 0.22b	30.3 ± 0.21a	N.D.	71.7 ± 0.43a
*p-*hydroxybenzoic acid	CK	1.1 ± 0.05a	2.6 ± 0.04b	13.4 ± 0.1e	3.66 ± 0.09b
	CAE	2.0 ± 0.01b	2.2 ± 0.01c	9.0 ± 0.1c	4.2 ± 0.02c
	XAE	1.0 ± 0.02a	2.7 ± 0.02a	11.8 ± 0.07d	3.7 ± 0.04a
	GAE	2.6 ± 0.05c	3.1 ± 0.01d	6.0 ± 0.01b	5.7 ± 0.11d
	CEAE	2.9 ± 0.03d	4.0 ± 0.03e	0.3 ± 0.01a	6.9 ± 0.06e
Caffeic acid	CK	N.D.	4.9 ± 0.07a	12.1 ± 0.11b	4.9 ± 0.07a
	CAE	N.D.	5.8 ± 0.31a	11.1 ± 0.05b	5.8 ± 0.31a
	XAE	N.D.	5.0 ± 0.07a	14.5 ± 0.13b	5.0 ± 0.07a
	GAE	N.D.	8.3 ± 0.01b	8.3 ± 0.07a	8.3 ± 0.01b
	CEAE	N.D.	9.1 ± 0.03b	7.7 ± 0.05a	9.1 ± 0.03b
Rutin	CK	1.8 ± 0.02a	N.D.	5.8 ± 0.11d	1.8 ± 0.02a
	CAE	2.0 ± 0.07b	N.D.	3.5 ± 0.08b	2.0 ± 0.07b
	XAE	1.8 ± 0.04a	N.D.	4.3 ± 0.02c	1.8 ± 0.04a
	GAE	0.5 ± 0.01b	N.D.	3.3 ± 0.11b	0.5 ± 0.01b
	CEAE	0.2 ± 0.02c	N.D.	2.7 ± 0.06a	0.2 ± 0.02c
*p*-Coumaric acid	CK	15.8 ± 0.21a	9.4 ± 0.29a	136.8 ± 0.89d	25.2 ± 0.50a
	CAE	21.4 ± 0.09a	11.6 ± 0.17b	124.4 ± 1.61c	32.9 ± 0.31b
	XAE	14.4 ± 0.07a	9.3 ± 1.21a	148.3 ± 2.35d	23.7 ± 1.28a
	GAE	27.7 ± 0.05b	15.2 ± 0.67c	82.7 ± 2.01b	42.8 ± 1.01c
	CEAE	29.6 ± 0.08b	17.7 ± 0.18d	34.9 ± 1.01a	47.6 ± 0.63d
Isoquercitrin	CK	37.1 ± 0.43b	26.5 ± 0.53a	19.6 ± 0.23d	63.6 ± 0.56a
	CAE	41.2 ± 0.91c	27.7 ± 0.15b	10.3 ± 1.01c	68.9 ± 0.78b
	XAE	35.5 ± 0.16a	27.7 ± 0.45a	12.3 ± 0.97e	62.9 ± 0.08a
	GAE	20.8 ± 0.34d	31.2 ± 0.21b	6.7 ± 0.26b	52.0 ± 2.01c
	CEAE	18.6 ± 0.79e	32.3 ± 0.19b	5.6 ± 0.31a	51.0 ± 1.23d
Sinapic acid	CK	4.7 ± 0.09a	N.D.	5.6 ± 0.23d	4.7 ± 0.09a
	CAE	2.3 ± 0.03b	N.D.	8.3 ± 1.01c	2.3 ± 0.03b
	XAE	2.1 ± 0.01a	N.D.	6.2 ± 0.97e	2.1 ± 0.01a
	GAE	3.4 ± 0.02a	N.D.	8.5 ± 0.26b	3.4 ± 0.02a
	CEAE	1.5 ± 0.02d	N.D.	7.3 ± 0.31a	1.5 ± 0.02d
Ferulic acid	CK	N.D.	N.D.	10.5 ± 0.19d	N.D.
	CAE	N.D.	N.D.	8.8 ± 0.13c	N.D.
	XAE	N.D.	N.D.	9.9 ± 0.31c	N.D.
	GAE	N.D.	5.6 ± 0.19a	6.4 ± 0.08b	5.6 ± 0.19a
	CEAE	N.D.	7.5 ± 0.31b	5.6 ± 0.11a	7.5 ± 0.31b
Quercetin-3-*O*-*β*-d-xylopyranoside	CK	53.6 ± 1.21a	N.D.	N.D.	53.6 ± 1.21a
	CAE	59.5 ± 0.61b	N.D.	N.D.	59.5 ± 0.61b
	XAE	48.2 ± 0.34a	N.D.	N.D.	48.2 ± 0.34a
	GAE	61.3 ± 0.48c	N.D.	N.D.	61.3 ± 0.48c
	CEAE	72.9 ± 1.05d	N.D.	N.D.	72.9 ± 1.05d
Quercetin-3-*O*-*α*-l-arabinoside	CK	87.2 ± 2.67b	N.D.	11.4 ± 0.41c	87.2 ± 2.67b
	CAE	113.2 ± 3.01c	N.D.	17.4 ± 0.26d	113.2 ± 3.01c
	XAE	89.6 ± 0.29b	N.D.	10.5 ± 1.11c	89.6 ± 0.29b
	GAE	68.2 ± 2.31d	15.1 ± 1.01b	10.2 ± 0.07b	83.2 ± 3.32d
	CEAE	11.1 ± 1.61a	4.2 ± 0.08a	7.1 ± 0.03a	15.3 ± 1.57a
Avicularin	CK	258.1 ± 1.79b	N.D.	16.4 ± 0.11c	258.1 ± 1.79b
	CAE	245.6 ± 1.94c	N.D.	14.2 ± 0.14c	245.6 ± 1.94c
	XAE	249.4 ± 3.27b	N.D.	15.7 ± 0.23c	249.4 ± 3.27b
	GAE	71.2 ± 2.29c	1.0 ± 0.08a	10.3 ± 0.31b	72.3 ± 2.29c
	CEAE	17.8 ± 0.21a	1.2 ± 0.02a	6.3 ± 0.05a	18.9 ± 0.23a
Quercitrin	CK	107.1 ± 1.68b	N.D.	11.3 ± 0.01b	107.1 ± 1.68b
	CAE	118.1 ± 1.17c	N.D.	9.6 ± 0.02b	118.1 ± 1.17c
	XAE	97.1 ± 1.45a	N.D.	10.4 ± 0.03b	97.1 ± 1.45a
	GAE	123.8 ± 2.64d	N.D.	5.2 ± 0.01a	123.8 ± 2.64d
	CEAE	112.9 ± 2.15b	21.4 ± 0.37b	4.9 ± 0.01a	134.3 ± 2.52b
Quercetin	CK	34.8 ± 0.57a	39.0 ± 1.61a	113.1 ± 0.31e	73.8 ± 2.14a
	CAE	59.5 ± 0.21b	47.2 ± 1.00b	98.4 ± 2.19c	106.7 ± 1.21b
	XAE	37.6 ± 1.21a	35.2 ± 0.29a	109.3 ± 1.45d	72.8 ± 1.36a
	GAE	134.1 ± 0.21c	65.0 ± 1.15c	25.4 ± 1.23b	199.0 ± 1.36c
	CEAE	177.0 ± 2.03d	81.9 ± 1.29d	10.3 ± 0.07a	258.9 ± 3.32d
Kaempferol	CK	5.1 ± 0.02b	N.D.	N.D.	5.1 ± 0.02b
	CAE	6.0 ± 0.07c	N.D.	N.D.	6.0 ± 0.07c
	XAE	5.0 ± 0.02a	N.D.	N.D.	5.0 ± 0.02a
	GAE	7.0 ± 0.03c	N.D.	N.D.	7.0 ± 0.03c
	CEAE	11.2 ± 0.01d	N.D.	N.D.	11.2 ± 0.01d

CK, untreated; CAE, cellulase-assisted extraction; XAE, xylanase-assisted extraction; GAE, *β*-glucosidase-assisted extraction; CEAE, complex enzyme-assisted extraction. Values with different letters in each column are significant following different enzymatic treatment (*p* < 0.05). N.D. Not detected.

**Table 2:** Antioxidant activity of the free, soluble-conjugate, insoluble bound, and total phenolics fractions of guava leaves following different enzyme-assisted extraction.

Stage	Antioxidant Activity			
	Free	Soluble-Conjugate	Insoluble-Bound	Total Soluble
ABTS value	(mmol TE/g DM)			
CK	20.6 ± 1.1Aa	6.5 ± 0.4Ba	20.2 ± 0.2Aa	27.1 ± 1.4a
CAE	23.5 ± 0.5Ab	9.5 ± 1.6Cb	16.8 ± 0.9Bb	33.0 ± 2.0b
XAE	18.7 ± 1.0Aa	7.2 ± 0.4Ba	20.6 ± 1.5Aa	25.9 ± 1.4a
GAE	29.0 ± 0.78Ac	10.8 ± 0.4Bc	12.2 ± 0.5Bc	39.9 ± 1.1c
CEAE	36.8 ± 0.4Ad	17.7 ± 0.1Bd	7.4 ± 1.2Cd	55.5 ± 0.5d
DPPH value	(mmol TE/g DM)			
CK	15.4 ± 0.2Ba	8.0 ± 0.1Ca	21.3 ± 0.2Aa	23.4 ± 0.3a
CAE	17.6 ± 0.4Ab	11.5 ± 0.3Bb	18.2 ± 1.1Ab	29.1 ± 0.7b
XAE	15.5 ± 1.09Ba	7.3 ± 0.9Ca	22.5 ± 0.5Aa	22.8 ± 1.9a
GAE	26.2 ± 0.7Ac	13.6 ± 1.1Cc	18.4 ± 1.3Bc	39.7 ± 1.8c
CEAE	35.1 ± 0.04Ad	17.9 ± 1.2Bd	11.1 ± 1.0Cd	53.0 ± 1.3d
FARP value	(μmol Fe(II)SE/g DM)			
CK	83.7 ± 2.0Aa	43.6 ± 1.0Ca	56.5 ± 1.4Ba	127.3 ± 5.5a
CAE	94.8 ± 4.2Ab	49.7 ± 2.3Bb	50.5 ± 2.8Bb	144.4 ± 3.8b
XAE	79.5 ± 1.37Aa	41.6 ± 3.4Ca	54.2 ± 1.4Ba	121.0 ± 6.5a
GAE	111.8 ± 4.7Ac	60.0 ± 2.1Bc	37.9 ± 0.9Cc	171.8 ± 6.1c
CEAE	162.6 ± 2.5Ad	79.7 ± 3.8Bd	22.7 ± 1.3Cd	242.3 ± 7.6d

CK, untreated; CAE, cellulase-assisted extraction; XAE, xylanase-assisted extraction; GAE, *β*-glucosidase-assisted extraction; CEAE, complex enzyme-assisted extraction. Each value was expressed as mean ± standard deviation (*n* = 3). Values with different letters (within row in uppercase letters (A–C), within columns in lowercase letters (a–d)) are significantly different (*p* < 0.05).

**Table 3:** Regression equation, R^2^, LOD, LOQ, linear range, and recovery rate analysis results of all phenolics analytes.

Analytes	Regression Equation ^a^	R^2^	LOD ^b^ (μg/mL)	LOQ ^b^ (μg/mL)	Linear Range (μg/mL)	Recovery Rate (%, *n* = 4)
Gallic acid	Y = 3.56 × 10^7^X + 2.06 × 10^4^	0.9921	0.024	0.076	5.64–70.5	98.99
Chlorogenic acid	Y = 2.51 × 10^7^X + 1.93 × 10^4^	0.9931	0.046	0.065	0.5–50	99.13
*p*-hydroxybenzoic acid	Y = 1.67 × 10^7^X + 3.11 × 10^4^	0.9978	0.078	0.073	0.5–50	97.98
Caffeic acid	Y = 8.53 × 10^6^X + 1.48 × 10^4^	0.9989	0.009	0.021	0.5–50	100.01
Rutin	Y = 1.99 × 10^7^X + 5.37 × 10^4^	0.9989	0.019	0.053	6–75	99.02
*p*-Coumaric acid	Y = 6.97 × 10^7^X + 1.13 × 10^5^	0.9981	0.031	0.024	0.5–50	98.99
Isoquercitrin	Y = 1.24 × 10^7^X + 1.40 × 10^3^	0.9992	0.067	0.048	6–75	100.13
Sinapic acid	Y = 1.87 × 10^7^X + 2.93 × 10^4^	0.9991	0.078	0.017	0.5–50	95.31
Ferulic acid	Y = 4.68 × 10^7^X + 6.91 × 10^4^	0.9978	0.065	0.029	0.5–50	99.89
Quercetin-3-*O*-*β*-d-xylopyranoside	Y = 1.78 × 10^7^X + 1.40 × 10^4^	0.9989	0.039	0.054	6–75	99.78
Quercetin-3-*O*-*α*-l-arabinoside	Y = 1.48 × 10^7^X + 2.03 × 10^3^	0.9996	0.048	0.051	6–75	98.97
Avicularin	Y = 1.45 × 10^7^X + 1.96 × 10^3^	0.9997	0.051	0.023	6–75	99.86
Quercitrin	Y = 1.30 × 10^7^X + 5.98 × 10^3^	0.9978	0.024	0.018	6–75	99.13
Quercetin	Y = 1.74 × 10^7^X − 1.45 × 10^4^	0.9992	0.031	0.010	2–75	101.07
Kaempferol	Y = 8.83 × 10^7^X − 2.23 × 10^4^	0.9992	0.076	0.037	6–75	99.65

^a^ Y representing the peak area; X representing the standard concentration; ^b^ LOD, limit of detection (S/N = 3); LOQ, limit of quantification (S/N = 10).

## Abbreviations

GL, Guava leaves; DM, Dried mass; CK, untreated; CAE, Cellulase-assisted extraction; XAE, Xylanase-assisted extraction; GAE, *β*-glucosidase-assisted extraction; CEAE, Complex enzyme-assisted extraction. TP, Total phenolics, TF, Total flavonoids, FP, Free phenolics; SCP, Soluble-conjugate phenolics; IBP, Insoluble-bound phenolics; FF, Free flavonoids; SCF, Soluble-conjugate flavonoids; IBF, Insoluble-bound phenolics; TSP, Total soluble phenolics; TSF, Total soluble flavonoids; DPPH, 1,1-Diphenyl-2-picrylhydrazyl; ABTS, 2,2-Azino-bis (3-ethylbenzothiazoline-6-sulfonic acid) diammonium salt; FRAP, Ferric reducing antioxidant power; LOD, Limit of detection; LOQ, Limit of quantification; N, Nicked DNA; S, Supercoiled DNA.
